# Baseline low-density lipoprotein cholesterol predicts the benefit of adding ezetimibe on statin in statin-naïve acute coronary syndrome

**DOI:** 10.1038/s41598-021-87098-x

**Published:** 2021-04-05

**Authors:** Jihaeng Im, Erisa Kawada-Watanabe, Junichi Yamaguchi, Hiroyuki Arashi, Hisao Otsuki, Yuko Matsui, Haruki Sekiguchi, Shinya Fujii, Fumiaki Mori, Hiroshi Ogawa, Nobuhisa Hagiwara

**Affiliations:** 1 Department of Cardiology, The Heart Institute of Japan, Tokyo Women’s Medical University, 8-1, Kawada-cho, Shinjuku, Tokyo, 162-8666 Japan; 2 Department of Cardiology, Cardiovascular Center of Sendai, Miyagi, Japan; 3 Department of Cardiology, Yokohama Medical Center, Kanagawa, Japan

**Keywords:** Cardiology, Risk factors

## Abstract

We aimed to evaluate the effect of baseline low-density lipoprotein cholesterol (LDL-C) on the outcomes of patients with the acute coronary syndrome (ACS) receiving pitavastatin monotherapy or the combination of pitavastatin + ezetimibe. In the HIJ-PROPER study, 1734 ACS patients with dyslipidemia were randomly assigned to receive pitavastatin or pitavastatin + ezetimibe therapy. Statin-naïve participants (n = 1429) were divided into two groups based on the median LDL-C level (131 mg/dL) at enrollment. The primary endpoint was a composite of all-cause death, non-fatal myocardial infarction, non-fatal stroke, unstable angina, and ischemia-driven coronary revascularization. The median follow-up was 3.2 years. In the < 131 mg/dL group (n = 686), LDL-C changes were − 34.0% and − 49.8% in the pitavastatin monotherapy and pitavastatin + ezetimibe-treated groups (*P* < 0.0001), respectively; in the ≥ 131 mg/dL group (n = 743), LDL-C changes were − 42.9% and − 56.4% (*P* < 0.0001, respectively. Kaplan–Meier analyses revealed that the primary endpoint was not significantly different between the treatment groups for the < 131 mg/dL group, however, it was significantly lower in patients treated with pitavastatin + ezetimibe in the ≥ 131 mg/dL group (Hazard ratio = 0.72, 95% confidence interval = 0.56–0.91, *P* = 0.007, *P* value for interaction = 0.012). Statin-naïve ACS patients with baseline LDL-C < 131 mg/dL did not clinically benefit from pitavastatin + ezetimibe, while patients with baseline LDL-C ≥ 131 mg/dL treated with pitavastatin + ezetimibe showed better clinical results than those treated with pitavastatin monotherapy.

**Clinical Trial Registration**: Original HIJ PROPER study; URL: http://www.umin.ac.jp/ctr. Unique Identifier; UMIN000002742, registered as an International Standard Randomized Controlled Trial.

## Introduction

Favorable evidence of intensive lipid-lowering therapy using statin or non-statin agents on primary and secondary prevention of cardiovascular adverse events has been accumulating to date^1–7^. The Cholesterol Treatment Trialists’ (CTT) Collaboration showed that an absolute reduction in low-density lipoprotein cholesterol (LDL-C) levels of 1-mmol/dL (38.7 mg/dL) was associated with a 22% relative reduction of major vascular events irrespective of the effect of treatment on baseline LDL-C levels^8^. In recent era of the intensive lipid-lowering approach for the acute coronary syndrome (ACS), the importance of baseline LDL-C is still unclear. In a sub-analysis of the PROVE IT–TIMI 22 (Pravastatin or Atorvastatin Evaluation and Infection Therapy–Thrombolysis In Myocardial Infarction 22), the benefit of intensive lipid-lowering therapy with atorvastatin 80 mg over pravastatin 40 mg progressively decreased as the baseline LDL-C levels lowered in statin-naïve ACS patients^9^. Recently, in a meta-analysis, Navarese et al. reported that trials enrolling patients with lower baseline LDL-C levels (< 100 mg/dL) did not show any mortality benefit^10^.

Among some of the non-statin agents for intensive lipid-lowering, ezetimibe is one of the guidelines recommended agents. The Improved Reduction of Outcomes: Vytorin Efficacy International Trial (IMPROVE-IT) demonstrated adding ezetimibe to statin further reduced LDL-C levels in ACS patients and improved their clinical outcomes^5^. However, the Heart Institute of Japan-PRoper level of lipid lOwering with Pitavastatin and Ezetimibe in acute coRonary syndrome (HIJ-PROPER) study, a randomized controlled trial that tested the efficacy of intensive lipid-lowering therapy and compared it with conventional lipid-lowering therapy in patients with ACS, was unable to sufficiently demonstrate the effectiveness of adding ezetimibe to pitavastatin^11^. The notable differences between the two studies were the proportion of statin-naïve patients (IMPROVE-IT vs. HIJ-PROPER = 65% vs. 83%), early invasive strategy by the percutaneous coronary intervention (PCI) in the acute phase (70% vs. 95%), and the LDL-C level at the time of hospitalization (93.8 mg/dL vs. 135.2 mg/dL).

To date, the effect of adding ezetimibe to statins based on differences in baseline LDL-C values in statin-naïve ACS patients remains to be determined. The purpose of the present study was to clarify the impact of baseline LDL-C values on clinical outcomes in statin-naïve ACS patients receiving contemporary lipid-lowering monotherapy with pitavastatin or the combination of pitavastatin and ezetimibe.

## Methods

This study is a prespecified subanalysis of the HIJ-PROPER study^11^. Briefly, the HIJ-PROPER study was a multicenter, prospective, randomized, open-label, blinded endpoint trial with an active-control design comparing two lipid-lowering treatment strategies involving 19 Japanese hospitals. A total of 1734 patients with ACS were randomly assigned to the intensive lipid-lowering therapy (pitavastatin + ezetimibe) or the standard lipid-lowering therapy (pitavastatin monotherapy) groups between January 2010 and April 2013. The target LDL-C level was 70 mg/dL in the pitavastatin + ezetimibe therapy and between 90 and 100 mg/dL in the pitavastatin monotherapy-treated group. During the entire study period, the pitavastatin dose (1–4 mg/day) was adjusted to achieve the target LDL-C for each individual, along with the study protocol.

In the current analysis, we focused on baseline levels of LDL-C. To avoid the pretreatment effect of statins, we excluded 292 patients who were pre-treated with statins and 13 patients who were lost to follow-up. Finally, 1429 statin-naïve ACS patients were included in this study. Patients were divided into two groups according to the median LDL-C level at the time of enrollment: the < 131 mg/dL group and the ≥ 131 mg/dL groups. The analysis by quartile groups was also performed to examine the validity of the median's cut-off.

Patients' characteristics, including clinical background, medication, laboratory data, were compared between the < 131 mg/dL group and the ≥ 131 mg/dL group. Clinical outcomes in each group were examined comparing the two lipid-lowering treatment strategies: intensive lipid-lowering therapy using pitavastatin + ezetimibe, and standard lipid-lowering therapy using pitavastatin monotherapy. The primary endpoint was a composite of the first occurrence of adverse cardiovascular events, including all-cause death, non-fatal myocardial infarction, non-fatal stroke, unstable angina pectoris, or ischemia-driven revascularization with either percutaneous coronary intervention or coronary artery bypass grafting, which was the same as the original HIJ-PROPER study. Participants were followed up by hospital clinicians or by other general practitioners. We determined the incidence of endpoint events during scheduled follow-up visits up to at least 36 months.

The study protocol conforms to the 1975 Declaration of Helsinki's ethical guidelines, as reflected in a priori approval by the institutional review board of Tokyo Women’s Medical University or ethics committee of each participating medical center. This additional analysis was included in the original trial protocol. Written informed consent for trial enrollment was obtained from all patients.

### Statistical analysis

Baseline characteristics are presented according to the baseline LDL-C level at the time of enrollment. Continuous variables are reported as means ± standard deviation or median [interquartile range], and categorical data are reported as absolute values and percentages. Comparisons of normally distributed continuous data were performed using Welch's t-test, comparisons of non-normally distributed continuous data were conducted using the Mann–Whitney U test, and comparisons of categorical data were carried out using Pearson's chi-squared test. In the present study, both in the < 131 mg/dL group and the ≥ 131 mg/dL group, the time to the first occurrence of events was analyzed in the intensive and standard lipid-lowering therapy groups using the Kaplan–Meier method with the log-rank test. According to the lipid-lowering strategy, the standard Cox proportional hazard regression model was used to calculate hazard ratios (HRs) and 95% confidence intervals (CIs) for the endpoints. A *P* value of < 0.05 indicates statistical significance unless stated otherwise. The methods of statistical analysis are based on the methods in the original HIJ-PROPER study. An independent statistical data center (Data Research Section, Kondo P.P. Inc., Osaka, Japan) analyzed data using SPSS Statistics (Ver.26, IBM, Chicago, IL) and R: A Language and Environment for Statistical Computing (Ver. 3.6.3, R Foundation for Statistical Computing, Vienna, Austria) software.

## Results

**Figure 1:**
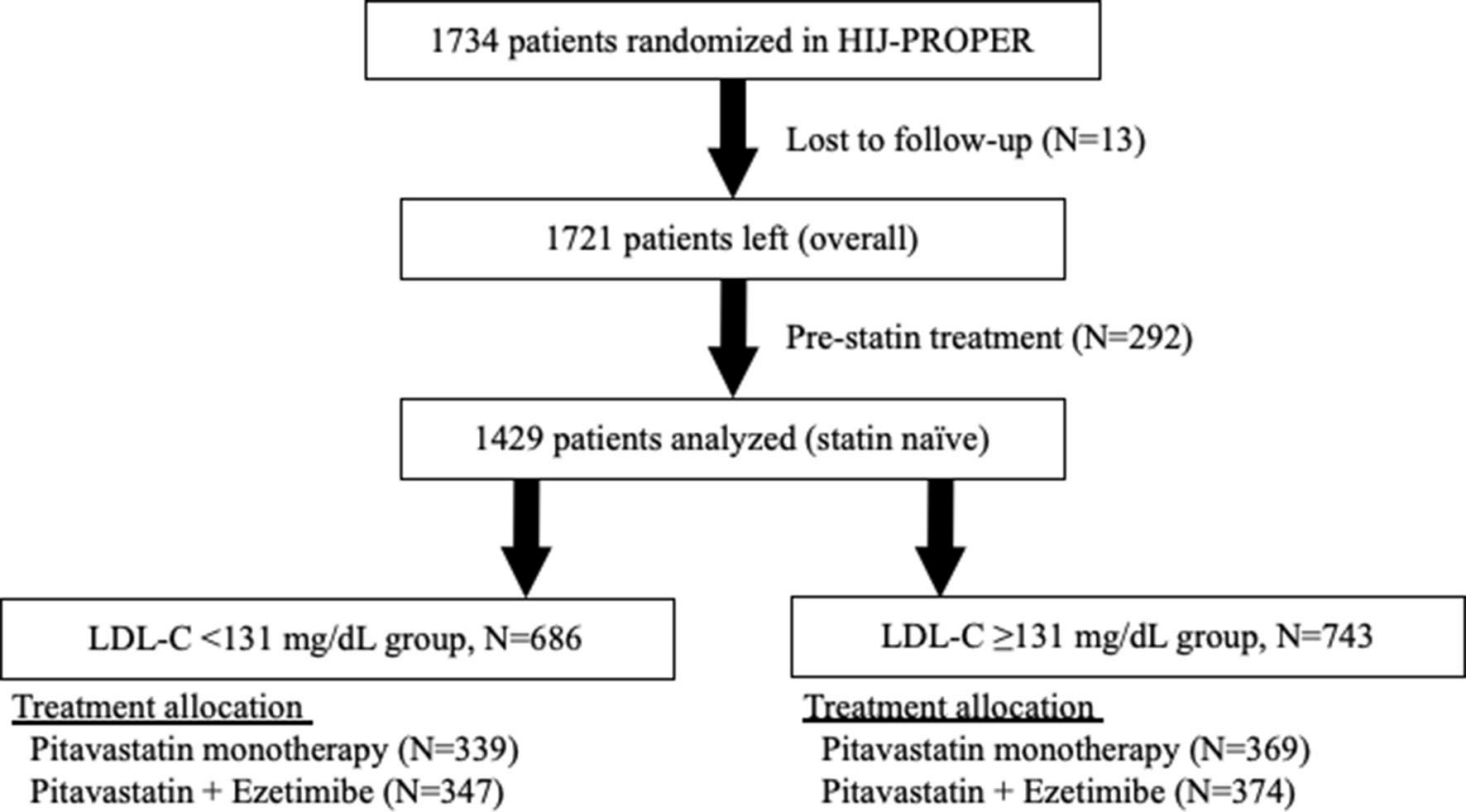
Study flow. LDL-C, low-density lipoprotein cholesterol.

Among the enrolled patients, 686 had baseline LDL-C levels < 131 mg/dL, while in 743 patients, baseline LDL-C levels were ≥ 131 mg/dL (Fig. 1). The distribution of baseline LDL-C levels in this patient subset is shown in Supplemental Figure 1.

The baseline clinical characteristics of the patients are shown in Table 1. Patients in the < 131 mg/dL group were older and mostly males, with a higher prevalence of hypertension; previous percutaneous coronary interventions/coronary artery bypass grafts; and also, higher prevalence of receiving beta-blockers, angiotensin-converting enzyme inhibitors/angiotensin II receptor blockers, and aspirin. In contrast, patients in the ≥ 131 mg/dL group showed a higher prevalence of current smoking and better renal function. In terms of cholesterol absorption/synthesis markers, the LDL-C ≥ 131 mg/dL group showed significantly higher levels of sitosterol, campesterol, and lathosterol (*P* < 0.0001, *P* < 0.007, and *P* < 0.0001, respectively). Baseline clinical characteristics according to the treatment allocation in each group and the quartile are shown in Supplemental Table 1 and 2.

**Table 1:** Baseline characteristics of the study population.

Variables†	Statin naïve; all patients (n = 1429)	LDL-C < 131 mg/dL group (n = 686)	LDL-C ≥ 131 mg/dL group (n = 743)	*P* value*
Age (years)	65.3 ± 11.9	67.4 ± 12.0	63.3 ± 11.5	< 0.0001
Male	1113 (77.9%)	555 (80.9%)	558 (75.1%)	0.01
BMI (kg/m^2^)	24.1 ± 3.5	23.9 ± 3.4	24.3 ± 3.5	0.06
Estimated GFR (mL/min/1.73 m^2^)	74.1 ± 25.9	72.3 ± 32.0	75.8 ± 18.5	< 0.0001
Hypertension	939 (65.7%)	474 (69.1%)	465 (62.6%)	0.01
Diabetes mellitus	398 (27.9%)	202 (29.5%)	196 (26.4%)	0.20
Current smoker	520 (36.4%)	218 (31.8%)	302 (40.7%)	< 0.0001
Previous myocardial infarction	76 (5.3%)	42 (6.1%)	34 (4.6%)	0.19
Previous revascularization	86 (6.0%)	53 (7.7%)	33 (4.4%)	0.009
**Type of index event**				0.20
STEMI	768 (53.7%)	356 (51.9%)	412 (55.5%)	
Non-STEMI	155 (10.8%)	71 (10.4%)	84 (11.3%)	
Unstable angina pectoris	506 (35.4%)	259 (37.8%)	247 (33.2%)	
**Medication**				
Beta-blockers	116 (8.1%)	76 (11.1%)	40 (5.4%)	< 0.0001
ACEI/ARB	338 (23.7%)	207 (30.2%)	141 (19.0%)	< 0.0001
Aspirin	176 (12.3%)	114 (16.6%)	62 (8.3%)	< 0.0001
**Cholesterol metabolism at baseline (mg/dL)**				
Total cholesterol	213 ± 35.5	187 ± 19.3	236 ± 31.2	< 0.0001
HDL-cholesterol	48.4 ± 12.4	47.8 ± 12.8	48.9 ± 11.9	0.02
LDL-cholesterol	138 ± 29.8	115 ± 10.1	159 ± 25.2	< 0.0001
Triglyceride	130 ± 71.0	123 ± 68.7	137 ± 72.4	< 0.0001
High-sensitivity CRP (mg/L)	8.51 [2.46, 26.1]	7.86 [2.37, 25.23]	9.24 [2.50, 27.05]	0.33
**Markers of cholesterol absorption/synthesis (µg/L)**				
Sitosterol	2.46 ± 1.57	2.18 ± 1.21	2.72 ± 1.80	< 0.0001
Lathosterol	1.96 ± 1.31	1.85 ± 1.19	2.06 ± 1.41	0.007
Campesterol	4.66 ± 2.40	4.16 ± 1.93	5.12 ± 2.69	< 0.0001

*P value refers to comparison between low LDL-C group and high LDL-C group.

Abbreviations: BMI, body mass index; GFR, glomerular filtration rate; STEMI, ST-elevation myocardial infarction; ACEI, angiotensin-converting enzyme inhibitors; ARB, angiotensin II receptor blockers; HDL, high-density lipoprotein; LDL-C, low-density lipoprotein cholesterol; CRP, C-reactive protein.

^**†**^Data are expressed as mean ± standard deviation, median [interquartile range], or as number (percentage).

**Figure 2:**
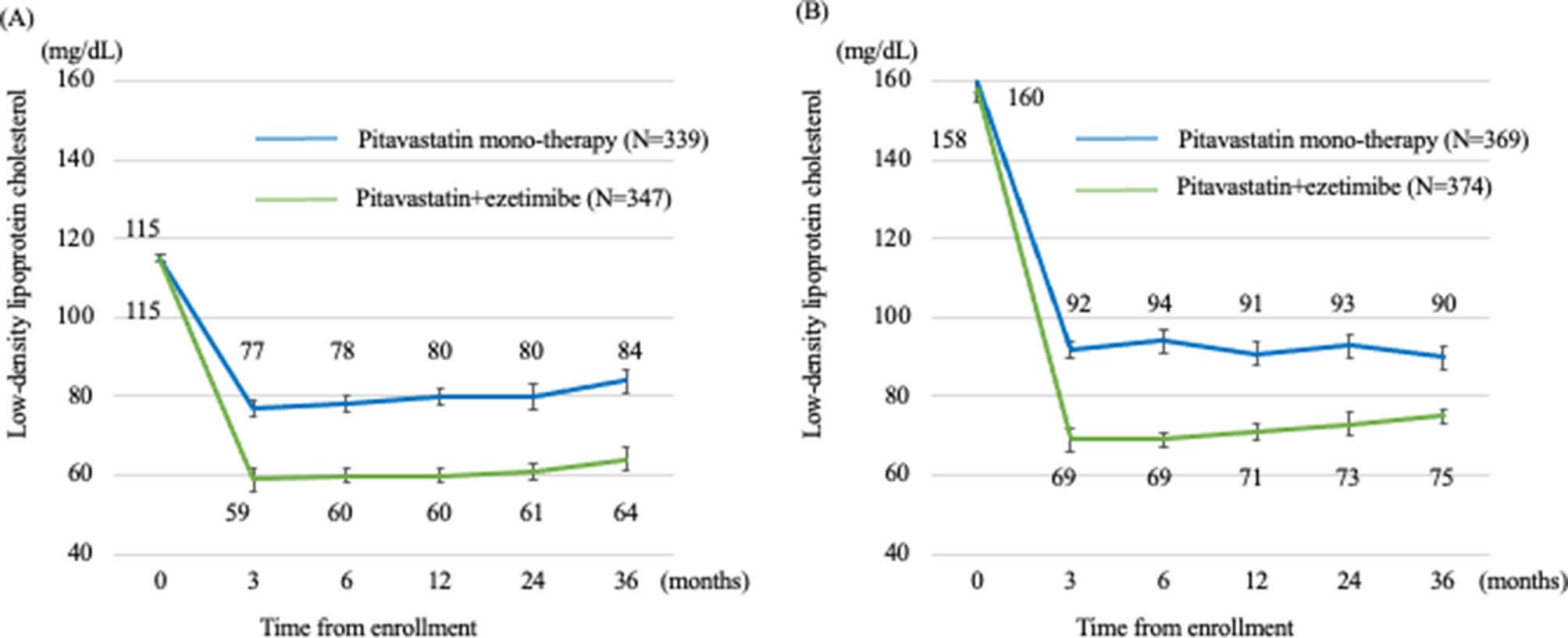
Changes in the mean LDL-C levels in the LDL-C < 131 mg/dL group (**A**) and the LDL-C < 131 mg/dL group (**B**). LDL-C, low-density lipoprotein cholesterol. LDL-C levels are expressed as mean ± 95% confidence intervals.

Figure 2 shows the changes in mean LDL-C levels in the LDL-C < 131 mg/dL group (Fig. 2A) and the LDL-C ≥ 131 mg/dL group (Fig. 2B) during the follow-up period. In the LDL-C < 131 mg/dL group, LDL-C changes were − 32.6 [95% confidence interval: 30.6, 34.6] % in the pitavastatin monotherapy group and − 49.0 [47.1, 50.9] % pitavastatin + ezetimibe-treated group (ANOVA *P* < 0.0001; LDL-C values at baseline and at the 3-month follow-up were 114.6 ± 9.8 and 76.9 ± 20.3 mg/dL in the pitavastatin monotherapy group and 114.7 ± 10.2 and 58.6 ± 19.7 mg/dL in the pitavastatin + ezetimibe group, respectively). In the LDL-C ≥ 131 mg/dL group, LDL-C changes were − 42.0 [40.5, 43.5] % and − 55.6 [54.2, 57.0] % respectively in the pitavastatin monotherapy group and in the pitavastatin + ezetimibe group (ANOVA *P* < 0.0001; LDL-C levels at baseline and 3 months in the pitavastatin monotherapy group were 159.7 ± 25.4 mg/dL and 91.5 ± 23.3 mg/dL, while in the pitavastatin + ezetimibe group, LDL-C levels were 158.3 ± 26.7 mg/dL and 69.4 ± 20.5 mg/dL, respectively) (Supplemental table 3).

**Figure 3:**
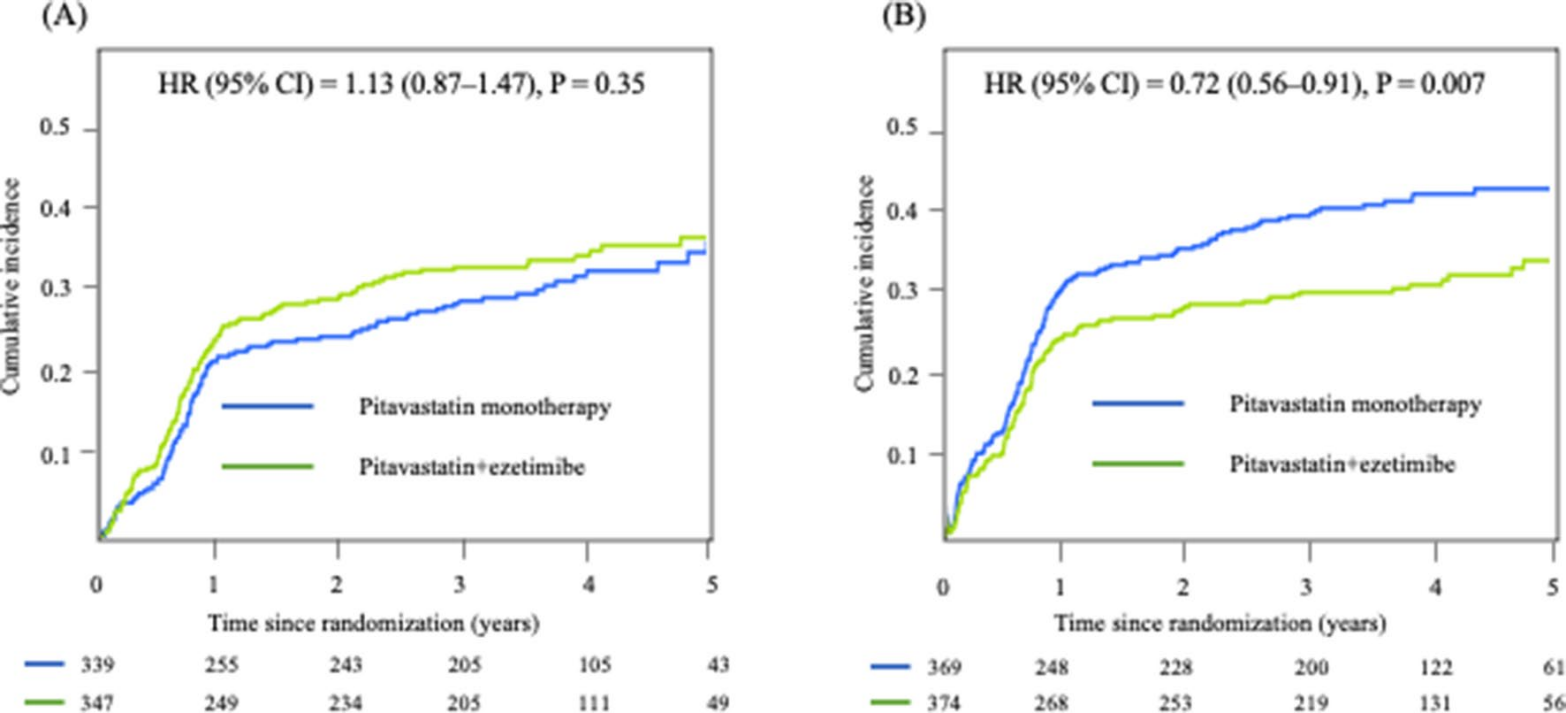
Primary outcome in the LDL-C < 131 mg/dL group (**A**) and the LDL-C < 131 mg/dL group (**B**). LDL-C, low-density lipoprotein cholesterol; HR, hazard ratio; CI, confidence interval.

**Figure 4:**
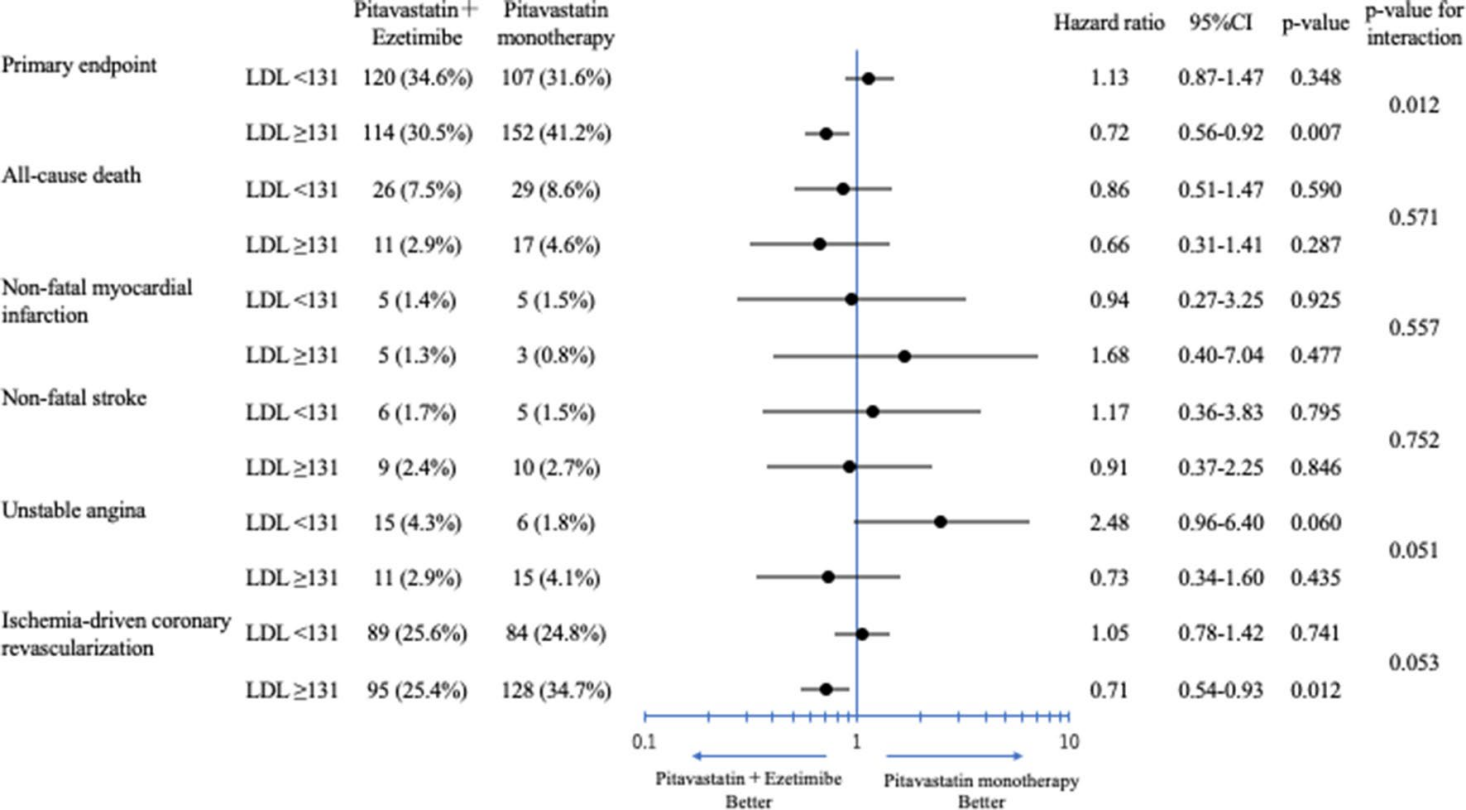
The hazard ratios for the primary endpoint and individual events stratified by median of baseline LDL-C. LDL-C, low-density lipoprotein cholesterol; CI, confidence interval.

The median observation period of this sub-analysis was 3.2 (interquartile range: 0.8, 4.3) years. Kaplan–Meier analyses revealed that the incidence of the primary endpoint was not statistically different between the two treatment groups in the LDL-C < 131 mg/dL group (HR = 1.13, 95% CI = 0.87–1.47, *P* < 0.35; Fig. 3A), while the incidence of the primary endpoint was significantly lower in patients treated with pitavastatin + ezetimibe than with pitavastatin monotherapy in the LDL-C ≥ 131 mg/dL group (HR = 0.72, 95% CI = 0.56–0.91, *P* = 0.007; Fig. 3B). There was significant heterogeneity in the treatment effect between the LDL-C < 131 mg/dL group and the LDL-C ≥ 131 mg/dL group for the primary endpoint (*P* value for interaction = 0.012, Fig. 4). The HRs for the primary endpoint stratified by quartiles of baseline LDL-C were shown in Supplemental figure 2. A decrease in the benefit of pitavastatin + ezetimibe therapy over pitavastatin monotherapy was seen from the highest to the lowest quartiles of baseline LDL-C. Besides, there was no significant interaction between treatment and continuous baseline LDL-C (HR [per 1 mg/dL increase of LDL-C], 1.001; 95%CI, 0.99–1.004, *P* value = 0.69).

## Discussion

The primary finding of the present study was that intensive therapy using pitavastatin + ezetimibe, compared with pitavastatin monotherapy, significantly improved clinical outcomes in the LDL-C ≥ 131 mg/dL group, but not in the LDL-C < 131 mg/dL group in statin-naïve ACS patients. Interestingly, the value of LDL-C 131 mg/dL is very close to the lower cut-off for borderline high classification (130–159 mg/dL) of LDL-C levels adopted by Adult Treatment Panel III^12^.

The phase Z of the A to Z trial, compared treatment effect between placebo + 20 mg of simvastatin vs. simvastatin 40/80 mg, showed no evidence of a greater relative treatment effect among patients with higher baseline levels of LDL-C^4^. In a sub-analysis of the PROVE IT–TIMI 22, the significant reduction in the hazards of the primary endpoints, including death, myocardial infarction (MI), unstable angina, revascularization after 30 days, and stroke, observed only in patients within the highest quartile of baseline LDL-C (median baseline LDL-C = 148 mg/dL) treated with atorvastatin 80 mg^9^. ODYSSEY OUTCOMES's subgroup analysis revealed that the treatment benefit on death after index ACS appeared to patients with a baseline LDL-C level of 100 mg/dL or greater, mainly^13,14^. In terms of the IMPROVE-IT trial, the mean LDL-C level at the time of hospitalization for the index event was 93.8 mg/dL. The baseline LDL-C level was much lower than that in the present study (137.7 mg/dL). Unfortunately, a subanalysis to examine the effect of baseline LDL-C level on the clinical outcome from the IMPROVE-IT trial is not available for the moment. Accordingly, to the best of our knowledge, this is the first report suggesting that the effects of adding ezetimibe treatment to a statin in statin-naïve ACS patients differ according to baseline LDL-C levels in the era of early invasive approach and comprehensive and modern medical treatment for high-risk atherosclerotic disease. A recent trial-level meta-analysis of 34 randomized clinical trials, including 270,288 heterogeneous patients from the early-1990s to the mid-2010s with various intensive treatment approaches using high-dose statins or non-statin agents like ezetimibe and PCSK-9 inhibitors, demonstrated that a more significant benefit from LDL-C–lowering therapy may occur for patients with higher baseline LDL-C levels^10^. The magnitude of benefit for all-cause mortality, cardiovascular disease mortality, and cardiovascular events appeared to decrease as the mean LDL-C levels of patients lowered. The mortality benefit was reduced by 9% for every 40 mg/dL decrease in the average baseline LDL-C of enrolled patients. The analysis also found that enrolled patients with lower baseline LDL-C levels (< 100 mg/dL) did not show a mortality benefit. Our present results in statin-naïve ACS patients suggest that the cut-off value of the baseline LDL-C level, predicting the efficacy of intensive lipid-lowering therapy prognosis, would differ depending on the clinical scenarios, which is consistent with the previous report of the impact of intensive statin therapy by Giraldez et al.^9^.

Besides, in statin-naïve ACS patients whose baseline LDL-C was < 131 mg/dL, no event suppression effect with intensive therapy using pitavastatin + ezetimibe was observed, even though LDL-C level of < 70 mg/dL was achieved as per the previous guideline-recommended target in the US^15^ and the suggested goal for people at high cardiovascular risk in the ESC/ESA guideline^16,17^. Conversely, there was an event suppression effect in patients whose baseline LDL-C was 131 mg/dL or more, with the achieved LDL-C value around the 70 mg/dL mark (69.4 ± 20.5 mg/dL at 3 months).

One possible explanation for why ezetimibe might be effective only in patients with a high baseline LDL-C level might be attributed to its role in inhibiting lipid absorption. In this study, the baseline levels of cholesterol absorption/synthesis markers were significantly higher in the LDL-C ≥ 131 mg/dL group than in the LDL-C < 131 mg/dL group. Several reports have demonstrated that a high plant sterol concentration is atherosclerogenic and is associated with coronary artery disease^18–20^. Especially in terms of sitosterol, as we previously reported, it is plausible that ezetimibe might exert a more potent effect under conditions of enhanced LDL-C absorption^21^.

Recent AHA/ACC guidelines for the management of dyslipidemia^22^ suggested a percent LDL-C decrease equal to or more than 50% as an indicator of intensive lipid-lowering therapy without any target goal LDL-C value. A previous meta-analysis of 3 randomized controlled trials involving 13,937 patients showed that patients with LDL-C reduction < 50% had a higher risk of cardiovascular events than those with LDL-C reduction ≥ of 50%, regardless of the attained LDL-C levels. Also, in patients with the atherosclerotic cardiovascular disease treated with a statin, the percent LDL-C reduction provided incremental predictive value for both statin dose and the achieved LDL-C levels, while the actual LDL-C levels achieved did not^23^. We observed that the LDL-C decline rate in the LDL-C ≥ 131 mg/dL group was greater than 50% of the baseline only in the pitavastatin + ezetimibe-treated group (55.6%). Interestingly, in the LDL-C < 131 mg/dL group, the percent LDL-C reduction in the pitavastatin + ezetimibe therapy was 49.0%, which was very close to 50%. However, there was no significant difference in the incidence of adverse events in comparison with pitavastatin monotherapy. Our results suggest that the goal of intensive lipid-lowering therapy should not be a target LDL-C level but a percent reduction of LDL-C ≥ 50% only in statin-naïve ACS patients with high baseline LDL-C values, since in patients with a low baseline LDL-C level a percent reduction of 50% might not be sufficient to prevent adverse cardiovascular events in contemporary practice.

Among the primary endpoint components, intensive therapy by adding ezetimibe significantly reduced the incidence of ischemic-driven coronary revascularization in the LDL-C ≥ 131 mg/dL group, although the *P* value for interaction could not show significant heterogeneity in the treatment effect. The extent of coronary revascularization might be biased because HIJ-PROPER was an open-labeled study. However, several studies have revealed that the potential impact of adding ezetimibe to statins might stabilize the lipid-rich plaques through increasing the fibrous cap thickness^24^, or reduce the volume of the coronary plaque directly^25^. In statin-naïve ACS patients with high baseline LDL-C, these favorable effects might be enhanced.

Adding ezetimibe to statin is one of the best options for intensive lipid-lowering therapy, following the recommendation of recent guidelines for managing dyslipidemia. Our results in the present study suggest a strong association between baseline LDL-C levels and the benefits of intensive lipid-lowering therapy by ezetimibe in the high-risk ACS subset, and would contribute to practical decision-making based on individual patient risk profiles.

Several limitations of this study should be considered. First, this was a retrospective study based on a prospective study subgroup analysis, and we did not provide a post-hoc power calculation. In the present analysis, the interaction between treatment and LDL dichotomized at the median was significant, however the interaction between treatment and continuous LDL was not significant. Thus, "significant" interaction might only be an artifact of the cut-point, and the results would be hypothetical and should be interpreted with caution. Second, the follow-up period was relatively short (median of 3.4 years). Third, our study consisted entirely of Japanese patients with ACS, which could affect the generalizability of our findings to non-Japanese patients. Fourth, although the endpoint of this study was reviewed by an independent endpoint committee that was blinded to the study treatment, the fact that ischemia-driven revascularization might affect the results weaken the gravity of the present study. Moreover, the high-intensity statin treatment is now the gold standard for post-ACS patients. Further large-scale, prospective studies with high-intensity statin treatment are warranted to reinforce the clinical reliability of our findings.

## Conclusions

In statin-naïve ACS patients with baseline LDL-C < 131 mg/dL, adding ezetimibe to pitavastatin treatment did not improve clinical outcomes. Therefore, a more aggressive or alternative lipid-lowering approach or interventions on other modifiable risk factors (diabetes, etc.) might be warranted in this subset. Our findings showed that statin-naïve ACS patients with baseline LDL-C ≥ 131 mg/dL treated by adding ezetimibe to pitavastatin had better clinical outcomes than those treated with pitavastatin monotherapy. Different treatment combinations in ACS patients with dyslipidemia will need to be further explored to obtain a more personalized approach based on baseline LDL-C values for the management of this high-risk subset.

## Supplementary Information

Supplementary Information.
