# Supplementary material for: Baseline low-density lipoprotein cholesterol predicts the benefit of adding ezetimibe on statin in statin-naïve acute coronary syndrome
Source: Sci Rep. 2021 Apr 5;11:7480. doi: 10.1038/s41598-021-87098-x (PMC8021554; doi:10.1038/s41598-021-87098-x)

Supplementary materials

Supplemental table 1-3, and Supplemental figure 1, 2

Baseline low-density lipoprotein cholesterol predicts the benefit of adding ezetimibe on statin in statin-naïve acute coronary syndrome

Jihaeng Im, MD[a,b]; Erisa Kawada-Watanabe, MD[a]; Junichi Yamaguchi, MD[a*]; Hiroyuki Arashi, MD[a]; Hisao Otsuki, MD[a]; Yuko Matsui, MD[a,c]; Haruki Sekiguchi MD[a]; Shinya Fujii MD[b]; Fumiaki Mori, MD[c]; Hiroshi Ogawa, MD[a]; Nobuhisa Hagiwara, MD[a];

Supplemental table 1. The baseline clinical characteristics according to the treatment allocation in LDL-C <131 group and LDL-C ≥131 mg/d group

Variable	LDL-C <131 mg/dL group			LDL-C ≥131 mg/dL group		
	Pitavastatin monotherapy (N=339)	Pitavastatin plus ezetimibe (N=347)	*p*-value	Pitavastatin monotherapy (N=369)	Pitavastatin plus ezetimibe (N=374)	*p*-value
Age (years)	67.5±12.1	67.4±11.9	0.87	63.2±11.6	63.4±11.5	0.85
Male	283(83.5%)	272 (78.4%)	0.09	277 (75.1%)	281 (75.1%)	0.98
BMI (kg/m^2)	24.0±3.4	23.9±3.5	0.62	24.1±3.6	24.6±3.5	0.049
Estimated GFR (mL/min/1.73m^2)	73.0±41.3	71.6±18.9	0.57	76.4±19.0	75.1±17.9	0.34
Hypertension	231 (68.1%)	243 (70.0%)	0.59	228 (61.8%)	237 (63.4%)	0.66
Diabetes mellitus	95 (28.0%)	108 (31.1%)	0.37	107 (29.0%)	90 (24.1%)	0.13
Current smoker	102 (30.1%)	116 (33.4%)	0.35	151 (40.9%)	151 (40.4%)	0.88
Previous myocardial infarction	19 (5.6%)	23 (6.6%)	0.58	21 (5.7%)	13 (3.5%)	0.15
Previous revascularization	26 (7.7%)	27 (7.8%)	0.96	20 (5.4%)	13 (3.5%)	0.20
Type of index event			0.95			0.13
STEMI	178 (52.5%)	178 (51.3%)		218 (59.1%)	194 (51.9%)	
Non-STEMI	35 (10.3%)	36 (10.4%)		40 (10.8%)	44 (11.8%)	
Unstable angina pectoris	126 (37.2%)	133 (38.3%)		111 (30.1%)	136 (36.4%)	
Medication						
Beta blocker	39 (11.5%)	37 (10.7%)	0.73	17 (4.6%)	23 (6.2%)	0.35
ACEIs/ARBs	103 (30.4%)	94 (27.1%)	0.34	67 (18.2%)	74 (19.8%)	0.57
Aspirin	55 (16.2%)	59 (17.0%)	0.78	33 (8.9%)	29 (7.8%)	0.56
Cholesterol metabolism						
Total cholesterol	187±19.8	188±18.7	0.51	237±30.8	235±31.5	0.34
LDL-cholesterol (mg/dL)	115±9.8	115±10.2	0.85	160±25.4	158±26.7	0.48
HDL-cholesterol (mg/dL)	47.4±12.7	48.3±12.8	0.35	48.9±11.8	49.0±12.0	0.86
Triglyceride (mg/dL)	123±69.2	123±68.3	0.96	139±76.1	134±68.5	0.35
High-sensitivity CRP (mg/L)	7.81 [2.06, 25.60]	7.87 [2.75, 25.00]	0.74	8.84 [2.70, 24.00]	10.60 [2.25, 29.63]	0.44
Markers of cholesterol absorption						
Sitosterol (μg/L)	2.20±1.29	2.15±1.12	0.56	2.68±1.56	2.76±2.03	0.56
Lathosterol (μg/L)	1.91±1.30	1.79±1.08	0.21	2.05±1.39	2.07±1.42	0.87
Campesterol (μg/L)	4.25±2.04	4.08±1.82	0.25	5.09±2.57	5.16±2.80	0.73

Abbreviations: BMI, body mass index; GFR, glomerular filtration rate; STEMI, ST-elevation myocardial infarction; ACEI, angiotensin-converting enzyme inhibitors; ARB, angiotensin II receptor blockers; *HDL,* high-density lipoprotein; LDL-C, low-density lipoprotein cholesterol; CRP, C-reactive protein.

Data are expressed as mean ± SD or median [interquartile rage], or as number (percentage)

Supplemental table 2. The baseline clinical characteristics according to the quartile of baseline LDL-C

	Quartile 1<br>LDL-C <116<br>(N=342)	Quartile 2<br>116≤ LDL-C < 131<br>(N=344)	Quartile 3<br>131≤ LDL-C < 153<br>(N=382)	Quartile 4<br>153≤ LDL-C<br>(N=361)	p-value
Age (years)	67.7±11.8	67.2±12.3	63.7±11.7	62.8±11.3	<0.0001
Male	283 (82.3%)	272 (79.1%)	297 (77.8%)	261 (72.3%)	0.01
BMI (kg/m^2)	24.1±3.5	23.7±3.3	24.3±3.6	24.4±3.5	0.08
Estimated GFR (mL/min/1.73m^2)	71.6±20.5	72.9±40.3	77.0±18.6	74.4±18.3	0.03
Hypertension	241 (70.5%)	233 (67.7%)	247 (64.7%)	218 (60.4%)	0.03
Diabetes mellitus	108 (31.6%)	94 (27.3%)	102 (26.7%)	94 (26.0%)	0.36
Current smoker	108 (31.6%)	110 (32.0%)	154 (40.3%)	148 (41.0%)	0.01
Previous myocardial infarction	22 (6.4%)	20 (5.8%)	17 (4.5%)	17 (4.7%)	0.6
Previous revascularization	24 (7.0%)	29 (8.4%)	18 (4.7%)	15 (4.2%)	0.06
Type of index event					0.12
STEMI	180 (52.6%)	176 (51.2%)	199 (52.1%)	213 (59.0%)	
Non-STEMI	40 (11.7%)	31 (9.0%)	42 (11.0%)	42 (11.6%)	
Unstable angina pectoris	122 (35.7%)	137 (39.8%)	141 (36.9%)	106 (29.4%)	
Medication					
Beta blocker	32 (9.4%)	44 (12.8%)	28 (7.3%)	12 (3.3%)	<0.0001
ACEIs/ARBs	109 (31.9%)	88 (25.6%)	90 (23.6%)	51 (14.1%)	<0.0001
Aspirin	55 (16.1%)	59 (17.2%)	39 (10.2%)	23 (6.4%)	<0.0001
Cholesterol metabolism					
Total cholesterol (mg/dl)	178±17.2	197±16.8	216±16.7	257±29.2	<0.0001
HDL-cholesterol (mg/dl)	48.2±13.0	47.5±12.6	48.5±12.2	49.3±11.7	0.25
LDL-cholesterol (mg/dl)	106±6.1	122±4.4	141±6.7	179±24.6	<0.0001
Triglyceride (mg/dl)	118±67.3	128±69.9	132±63.7	142±70.7	0.0002
High-sensitivity CRP (mg/l)	8.14<br>[2.49, 25.5]	7.24<br>[2.06, 24.93]	9.42<br>[2.49, 26.83]	9.16<br>[2.61, 27.60]	0.96
Markers of cholesterol absorption/synthesis (μg/L)					
Sitosterol	2.07±1.07	2.28±1.33	2.48±1.29	2.97±2.20	<0.0001
Lathosterol	1.76±1.11	1.94±1.26	1.97±1.33	2.15±1.32	0.001
Campesterol	4.02±1.74	4.32±2.10	4.79±2.28	5.48±3.02	<0.0001

Abbreviations: BMI, body mass index; GFR, glomerular filtration rate; STEMI, ST-elevation myocardial infarction; ACEI, angiotensin-converting enzyme inhibitors; ARB, angiotensin II receptor blockers; *HDL,* high-density lipoprotein; LDL-C, low-density lipoprotein cholesterol; CRP, C-reactive protein.

Data are expressed as mean ± SD or median [interquartile rage], or as number (percentage)

Supplemental table 3. Changes in the LDL-C levels at baseline and 3 months after therapy

	Baseline	3-month	*p* value	% change*	*p* value**
LDL-C <131 mg/dL group					
Pitavastatin mono-therapy	114.6 ± 9.8	76.9 ± 20.3	<0.0001	32.6 [30.6, 34.6]	
Pitavastatin+ezetimibe therapy	114.7 ± 10.2	58.6 ± 19.7	<0.0001	49.0 [47.1, 50.9]	<0.0001
LDL-C ≥131 mg/dL group					
Pitavastatin mono-therapy	159.7 ± 25.4	91.5 ± 23.3	<0.0001	42.0 [40.5, 43.5]	
Pitavastatin+ezetimibe therapy	158.3 ± 26.7	69.4 ± 20.5	<0.0001	55.6 [54.2, 57.0]	<0.0001

Abbreviations: LDL-C, low-density lipoprotein cholesterol;

Data are expressed as mean ± standard deviation

*% change from baseline to 3-month follow-up are expressed as mean [95% confidence interval]

***p*-value refers to the difference in the percent change between pitavastatin mono-therapy and pitavastatin+ezetimibe therapy.

Supplemental figure 1. Distribution of LDL-C in statin naïve patients (N = 1429)

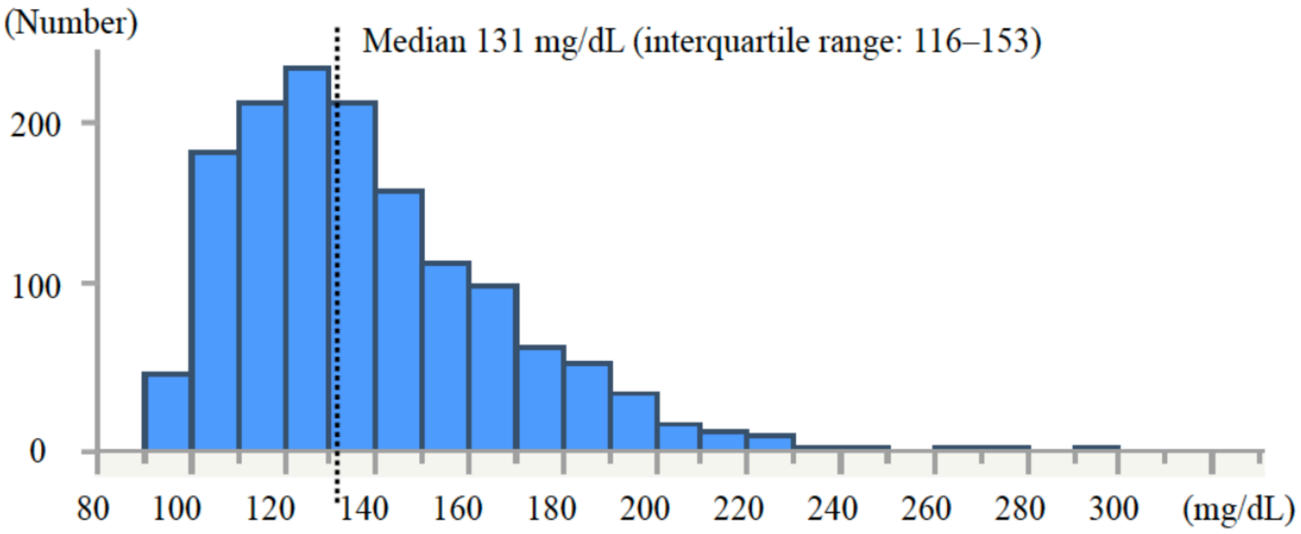

Supplemental figure 2. The hazard ratios for the primary endpoint stratified by quartiles of baseline LDL-C

Quartile	Baseline LDL-C (mg/dL)	Pitavastatin + Ezetimibe	Pitavastatin monotherapy	Primary endpoint	Hazard ratio	95%CI	p-value	p-value for interaction (vs. Quartile 4)
1	LDL-C <116	60 (35.1%)	54 (31.6%)		1.14	0.79-1.65	0.475	0.013
2	116≤ LDL-C < 131	60 (34.1%)	53 (31.5%)		1.11	0.77-1.61	0.568	0.017
3	131≤ LDL-C < 153	70 (35.4%)	76 (41.3%)		0.83	0.60-1.15	0.261	0.169
4	153≤ LDL-C	44 (25.0%)	76 (41.1%)		0.59	0.41-0.86	0.006	
	**overall**	**234 (32.5%)**	**259 (36.6%)**		**0.89**	**0.74-1.06**	**0.181**	

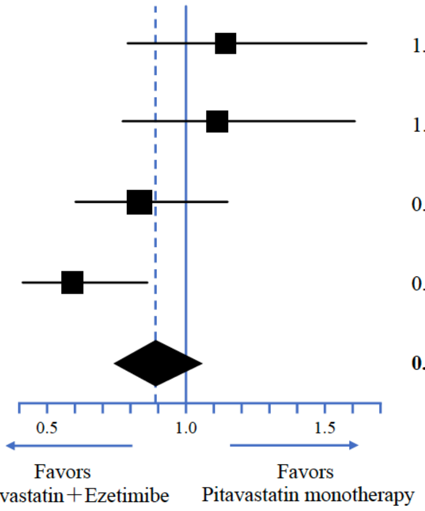

Supplement: Supplementary file 1 — Supplementary Information. [file 41598_2021_87098_MOESM1_ESM.pdf]
